# Modern contraceptive utilization and its associated factors among married women in Senegal: a multilevel analysis

**DOI:** 10.1186/s12889-021-10252-7

**Published:** 2021-01-28

**Authors:** Betregiorgis Zegeye, Bright Opoku Ahinkorah, Dina Idriss-Wheeler, Comfort Z. Olorunsaiye, Nicholas Kofi Adjei, Sanni Yaya

**Affiliations:** 1 HaSET Maternal and Child Health Research Program, Shewarobit Field Office, Shewarobit, Ethiopia; 2 School of Public Health, Faculty of Health, University of Technology Sydney, Ultimo, Australia; 3 Interdisciplinary School of Health Sciences, University of Ottawa, Ottawa, Ontario Canada; 4 Department of Public Health, Arcadia University, Glenside, PA USA; 5 Leibniz Institute for Prevention Research and Epidemiology, BIPS, Bremen, Germany; 6 School of International Development and Global Studies, University of Ottawa, 120 University Private, Ottawa, ON K1N 6N5 Canada; 7 The George Institute for Global Health, Imperial College London, London, UK

**Keywords:** Contraceptive utilization, Modern contraceptive, Senegal; sexual and reproductive health

## Abstract

**Background:**

Utilization of modern contraceptives is still low in low-and middle-income countries, although fertility and population growth rates are high. In Senegal, modern contraceptive utilization is low, with few studies focusing on its associated factors. This study examined modern contraceptive use and its associated factors among married women in Senegal.

**Methods:**

Data from the 2017 Continuous Demographic and Health Survey (C-DHS) on 11,394 married women was analysed. We examined the associations between the demographic and socioeconomic characteristics of women and their partners and modern contraceptive use using multilevel logistic regression models. Adjusted odds ratios with 95% confidence intervals (CI) were estimated.

**Results:**

The utilization of modern contraceptives among married women was 26.3%. Individual level factors associated with modern contraceptive use were women’s age (45–49 years-aOR = 0.44, 0.30–0.63), women’s educational level (higher-aOR = 1.88, 1.28–2.76) husband’s educational level (higher-aOR = 1.43, 1.10–1.85)), number of living children (5 or more children-aOR = 33.14, 19.20–57.22), ideal number of children (2 children-aOR = 1.95, 1.13–3.35), desire to have more children (wants no more-aOR = 2.46, 2.06–2.94), ethnicity (Diola-aOR = 0.70, 0.50–0.99), media exposure (yes-aOR = 1.44, 1.16–1.79)), wealth index (richer-aOR = 1.31, 1.03–1.67) and decision making power of women (decision making two-aOR = 1.20, 1.02–1.41). Whereas, region (Matam-aOR = 0.35, 0.23–0.53), place of residence (rural-aOR = 0.76, 0.63–0.93), community literacy level (high-aOR = 1.31, 1.01–1.71) and community knowledge level of modern contraceptives (high-aOR = 1.37, 1.13–1.67) were found as significant community level factors.

**Conclusions:**

The findings indicate that both individual and community level factors are significantly associated with modern contraceptive use among married women in Senegal. Interventions should focus on enhancing literacy levels of women, their husbands and communities. Furthermore, strengthening awareness and attitude towards family planning should be given priority, especially in rural areas and regions with low resources.

## Background

Family planning has been associated with several benefits such as reduction in maternal and infant mortality [1–4]. This can generally be achieved by encouraging girls’ education [2, 4], and enhancing female labor force participation [4–7]. In developing countries, contraceptive use prevented 218 million unintended pregnnacies in 2012, averting 55 million unplanned births, 138 million abortions (of which 40 million were unsafe), 25 million miscariages and 118,000 maternal deaths [2]. In 2017, about 63% of married women of reproductive age or their partners used either modern or traditional methods of contraception [5, 8]. However, compared to other continents, there is low prevalence of contraceptive use in Africa (36%). For example, in 2017, contraceptive prevalence was 58% in Oceania while a prevalence of 75% was recorded in the Caribbean, Northern and Latin America [5, 8]. Although there have been improvements in the use of contraceptives from 8% in 1970 to 36% in 2017 in Africa, there are still significant gaps in relation to the utilization of modern contraceptives among couples who have the intention to delay or prevent pregnancy [5, 8].

Senegal is a one of the African countries with a very high fertility rate and rapid population growth [9, 10]. There are variations in total fertility rate (TFR) from 6.35 children per woman in rural areas to 3.9 per woman in urban areas [7]. Senegal is also one of the countries with lowest family planning utilization of 28% as at 2017 [5, 8]. Higher proportion of women are not getting family planning services, as the unmet need is estimated to be 29%. That means, approximately one married women of every three women who has the intention to stop giving birth or delay her subsequent pregnancy is not using contraceptive. Unmet need was also higher (32%) in Dakar region, where 25% of the country’s population live [7, 11].

There have been several studies on factors that influence contraceptive utilization in Africa [6, 12–19]. These studies have identified several individual and community-level factors such as age, marital status, ethnicity, religion, educational level, wealth index, place of residence and region as factors associated with moden contraceptive use [6, 12–19]. In terms of education, studies have shown that utilization of modern contraceptives is connected to partner’s educational level [13, 20]. Other studies suggest that husband’s educational level may influence contraceptive use for family planning. Closely connected to education are findings that increased communication between couples facilitate support of contraceptive utilization by a husband [20, 21]. Other studies have found associations between media exposure and contraceptive use [22]. The media has been found as beneficial in raising awareness on health issues within communities, as well as addressing social and cultural issues [23]. In terms of wealth, studies have found that the odds of contraceptive use is higher among richer women as compared to poorest [24–26] and this has been linked to the influence of socioeconomic status [25, 26]. Married women with living children have been found to have higher odds of modern contraceptive use compared to married women with no living children [6, 27, 28] similar to those who live in urban areas [17, 29]. The association between ethnicity and contraceptive utilization [30] has been attributed to unequal access of public services to people lower in the ethnic hierarchy, illiteracy, and poor socio-economic conditions among some ethnic groups [31–33].

In Senegal, despite the low contraceptive utilization, few studies have focused on factors associated with the low contraceptive usage [13, 17, 34–36]. Moreover, available studies on utilization of contraceptivs were either based on the 2012/13 DHS [35] and 1992–2013/14 DHS data [13], focused on single factor [13, 34] and limited to a single study area [34, 36]. None of these studies have examined the association between individual and community-level factors and utilization of contraceptives in Senegal. Therefore, the present study aimed to examine the prevalence and factors associated with the use of modern contraceptives among married women in Senegal.

The findings of the study will inform existing policies aimed at enhancing the use of contraceptives in Senegal. Among the important policies in Senegal is the 2005 Reproductive Health (RH) Law, which is based on the right of individuals and couples to RH, equitable access to RH care, and respect for the physical integrity of women and girls [37, 38]. In addition, giving priorities for disadvantaged women or narrowing of disparities in the use of contraceptive is another strategy that exists in Senegal and the findings of the current study will influence the success of these strategies. Finally, the findings of the current study will also help to enhance the strategy of ensuring increase service uptake of contraceptive in Senegal through media exposure [39].

## Methods

### Study setting

Senegal, located in West Africa, is well-known as the “Entry to Africa” [40, 41]. Up to half of its 15.4 million people (2016) live in and around Dakar and other urban areas [40]. In Senegal, there is a hierarchical health system model, where regional medical officers oversee the activities of health districts in every region. Health districts are made up of a health centre and a linkage to rural health posts, often under the supervision of allied community-level facilities [42]. These health facilties are operated by community health workers [43]. In senegal, family planning usage has been low- only about 18% of women with partners aged 15–49 used a modern contraceptive method in 2013 [10]. In 2015, it increased marginally to 21.2%, and 21–23% had an unmet need-non-use of contraceptives among fecund and sexually active women who do not want any more pregnancy or have the intention to delay the next pregnancy [44], for family planning [45].

### Data source

Data from the 2017 Continuous-Demographic and Health Survey (C-DHS) [45] was used in this study. The C-DHS is a nationally representative data undertaken by the Senegal Agency of Statistics and Demography (ANSD) in collaboration with ICF International [45]. A two-stage stratified sampling procedure is adopted in selecting research participants. In the first phase, clusters/enumeration areas (EAs) are selected, guided by a sample frame developed during the 2013 census [45]. In all, 8800 households, made up of 4092 located in urban areas and 4708 in rural areas were considered in the survey [45]. For this study, a sample of 11,394 married women aged 15–49 with information on modern contraceptive use were considered.

### Variables

The main outcome variable of this study was modern contraceptive use. To derive this variable, married women were asked of the contraceptive methods they use. Those who mentioned that they used modern contraceptives were coded as modern contraceptive method users while those who used traditional/folkloric/no method were coded as non-modern contraceptive users. Details of the types of contraceptives considered as modern, traditional or folkloric have been published in previous studies [2, 4, 46]. Married women who were sterilized and declared as infecund were excluded from the analysis. Several individual and community-level explanatory variables were chosen based on prior evidence [6, 14–16, 18, 19, 36, 47–50].

### Individual level factors

These included demographic and socio-economic variables such as women’s age (15-19, 20-24, 25-29, 30-34, 35-39, 40-44, 45-49) women’s educational level (No formal education, primary school, secondary school, higher), husband/partner education (No formal education, primary school, secondary school, higher), number of living children (no children, 1–2 children, 3–4 children, five or more children), ideal number of children (no child, 1 child, 2 children, 3 children, 4 children, 5 children, 6 children and above and non-numeric response (e.g upto God/Allah) and desire for more children (wants within 2 years, wants after 2 years, wants but unsure of timing, undecided, wants no more). Others were distance to health facility (a big problem, not a big problem), ethnicity (Wolof, Poular, Serer, Mandingue/soc., Diola, Sonink, other Senegalese and other), religion (Muslim and Christian) and media exposure (newspaper, radio or television [TV]). Media exposure was derived from a combination of three variables; reading of newspaper or magazine, listening to radio and watching television. DHS grouped all of the three variables into three categories not at all (0), less than once a week (1) and at least once a week (2). We then, re-categorized these into two (binary) for all the three variables. Those who didn’t read newspaper/magazine, or listened to radio or watched television at all were coded as zero (0), and those who read newspaper/magazine, listened to radio or watched television for less than once a week and at least once a week were coded as one (1). We then generated, media exposure by adding the three variables. If the respondent had no exposure at all for all three variables we coded as ‘no’. If the respondent had exposure to at least one of the three variables, we coded as ‘yes’.

Other individual level variables included wealth index (poorest, poorer, middle, richer, richest), attitude towards wife beating (refuse versus accept) and women’s empowerment [51, 52]. Women empowerment as a variables was derived from respondent’s decision making capacity on own health care, large household purchases, and visits to family and relatives. Respondents whose responses to these three decision making variables showed that their decisions are made by their husband/partner alone or other were considered as having “no decision-making”. Respondents who mentioned that they took decisions alone or with their husband/partner on two of the decision making variables were put in the category “decision making one”. Finally, women who decided alone or with their partner/husband on all the three decision making variables were coded into “decision making two”.

### Community level factors

Place of residence (urban, rural), region (Dakar, Ziguinchor, Diourbel, Saint Louis, Tambacounda, Kaolack, Thies, Louga, Fatick, Kolda, Matam, Kaffrine, Kedougo and Sedhiou), community-level literacy-proportion of women who can read and write (low medium, high), community-level socioeconomic status-the proportion of women within the richest wealth quintile (low, medium, high) and community-level knowledge about modern contraceptive-the proportion of women with knowledge about modern contraceptives (low, medium, high) were considered as community-level variables.

### Statistical analysis

This study employed both descriptive statistics and multi-level logistic regression models in the data analyses. The descriptive analysis employed percentages to describe the study variables. This was followed by the use of chi-square test to show the independent associations between the individual and community level variables and modern contraceptive use using *p*-values < 0.05 as cut of points. Finally, multilevel logistic regression analysis made up of four models was carried out using the “melogit” stata command. Results were presented using adjusted odds ratios (aOR) at 95% confidence intervals (CI). The multilevel logistic regression models consisted of fixed effects (measures of association) and random effects (measures of variability) [19, 53, 54]. Muli-collinearity test was done among the independent variables using the variance inflation factor (VIF) and the results indicated no evidence of high collinearity among the explanatory variables (Mean VIF = 1.43, Minimum VIF = 1.01, Maximum VIF = 2.26). Previous studies have shown that VIF values less than 10 are tolerable [55–57]. Sample weights were employed and the ‘svyset’ command was used to correct for under and over-sampling [26, 58]. All analyses were carried out in stata 14 for windows.

### Ethical consideration

Data for this study was derived from the website of MEASURE DHS through a standard national survey. Permission to use the data was granted by MEASURE DHS review board. All data were anonymized prior to the authors receiving the data.

## Results

### Background characteristics of respondents and use of modern contraceptives

Out of the 11,394 married women, 26.3% were using modern contraceptives. Results on the distribution of modern contraceptive use across the explanatory variables show that most of the women who used modern contraceptives were aged 35–39 (29.62%), had higher level of education (36.68%), had husbands with higher level of education (34.83%), lived in urban areas (31.36%) and belonged to the Serer ethnic group (28.47%). The results further showed that most of the women who used modern contraceptives were Christians (217.61%), had richest wealth index (33.84%), had five or more children (32.75%), were against wife beating (27.62%), lived in Dakar (42.05%) and were exposed to media (24.19%). Further results also indicate high modern contraceptive use among women with 2 children (34.23%), those who considered distance to the health facility as not a big problem (25.13%), those who lived in high literacy level communities (33.47%), those who lived in communities with high socio-economic status (32.65%) and those who lived in communities where knowledge of modern contraceptives was high (28.94%). The results of the bivariate analysis indicate that all the explantatory variables had statistically significant associations with modern contraceptive use at *p* < 0.05 (Table 1).

**Table 1:** Utilization of modern contraceptives across explanatory variables Variables

Modern Contraceptive use	Non use	Use	Chi-square, ***P***-value
	Frequency/Percent	Frequency/Percent	
	8770 (73.69)	2624 (26.31)	
**Women’s age**			χ2 = 246.16, *p* < 0.001
15–19	963 (92.15)	82 (7.85)	
20–24	1566 (82.16)	340 (17.84)	
25–29	1706 (76.26)	531 (23.74)	
30–34	1598 (72.64)	602 (27.36)	
35–39	1181 (70.38)	497 (29.62)	
40–44	1004 (72.81)	375 (27.19)	
45–49	752 (79.24)	197 (20.76)	
**Women’s educational level**			χ2 = 100.18, *p* < 0.001
No formal education	5820 (79.79)	1474 (20.21)	
Primary school	1736 (72.12)	671 (27.88)	
Secondary school	1087 (72.81)	406 (27.19)	
Higher	126 (63.32)	73 (36.68)	
**Husband's educational level**			χ2 = 189.69, *p* < 0.001
No formal education	6703 (80.15)	1660 (19.85)	
Primary school	922 (66.71)	460 (33.29)	
Secondary school	825 (71.24)	333 (28.76)	
Higher	320 (65.17)	171 (34.83)	
**Place of residence**			χ2 = 269.61, *p* < 0.001
Urban	2946 (68.64)	1346 (31.36)	
Rural	5824 (82.01)	1278 (17.99)	
**Ethnicity**			χ2 = 88.21, *p* < 0.001
Wolof	2767 (74.91)	927 (25.09)	
Poular	3075 (81.24)	710 (18.76)	
Serer	1045 (71.53)	416 (28.47)	
Mandingue/soc	798 (77.63)	230 (22.37)	
Diola	263 (72.65)	99 (27.35)	
Sonink	149 (80.11)	37 (19.89)	
Other Senegalese	415 (73.19)	152 (26.81)	
Other	258 (82.96)	53 (17.04)	
**Religion**			χ2 = 3.60, *p* = 0.057
Muslim	8555 (77.09)	2542 (22.91)	
Christian	215 (72.39)	82 (27.61)	
**Wealth index**			χ2 = 251.21, *p* < 0.001
Poorest	2572 (82.94)	529 (17.06)	
Poorer	2218 (81.97)	488 (18.03)	
Middle	1934 (75.28)	635 (24.72)	
Richer	1215 (68.96)	547 (31.04)	
Richest	831 (66.16)	425 (33.84)	
**Number of living children**			χ2 = 525.33, *p* < 0.001
No child	1380 (98.29)	24 (1.71)	
1–2 children	2906 (79.79)	736 (20.21)	
3–4 children	2212 (71.89)	865 (28.11)	
Five or more children	2272 (69.46)	999 (30.54)	
**Decision making**			χ2 = 183.50, *p* < 0.001
No decision making	5918 (80.87)	1400 (19.13)	
Decision making one	1969 (71.26)	794 (28.74)	
Decision making two	883 (67.25)	430 (32.75)	
**Wife-beating**			χ2 = 95.52, *p* < 0.001
Accept	5361 (80.21)	1323 (19.79)	
Refuse	3409 (72.38)	1301 (27.62)	
**Region**			χ2 = 434.48, *p* < 0.001
Dakar	481 (57.95)	349 (42.05)	
Ziguinchor	352 (71.69)	139 (28.31)	
Diourbel	813 (83.30)	163 (16.70)	
Saint Louis	563 (73.02)	208 (26.98)	
Tambacounda	753 (85.67)	126 (14.33)	
Kaolack	559 (76.26)	174 (23.74)	
This	615 (66.13)	315 (33.87)	
Louga	694 (78.77)	187 (21.23)	
Fatick	617 (72.59)	233 (27.41)	
Kolda	683 (78.51)	187 (21.49)	
Matam	771 (89.24)	93 (10.76)	
Kaffrine	766 (77.45)	223 (22.55)	
Kedougou	520 (88.29)	69 (11.71)	
Sedhiou	583 (78.68)	158 (21.32)	
**Media exposure**			χ2 = 81.44, *p* < 0.001
No	964 (87.88)	133 (12.12)	
Yes	7806 (75.81)	2491 (24.19)	
**Desire for more children**			χ2 = 543.34, *p* < 0.001
Wants within 2 years	2898 (87.98)	396 (12.02)	
Wants after 2 years	3380 (71.93)	1319 (28.07)	
Wants, unsure timing	705 (89.92)	79 (10.08)	
Undecided	141 (79.66)	36 (20.34)	
Does not want	1379 (64.71)	752 (35.29)	
**Ideal number of children**			χ2 = 127.72, *p* < 0.001
0 child	164 (73.54)	59 (26.46)	
1 child	12 (80.00)	3 (20.00)	
2 children	98 (65.77)	51 (34.23)	
3 children	346 (75.22)	114 (24.78)	
4 children	1199 (71.84)	470 (28.16)	
5 children	1414 (73.49)	510 (26.51)	
6 children and above	3811 (77.35)	1116 (22.65)	
Non-numeric response	1726 (85.15)	301 (14.85)	
**Distance to health facility**			χ2 = 65.37, *p* < 0.001
Big problem	2824 (81.81)	628 (18.19)	
Not a big problem	5946 (74.87)	1996 (25.13)	
**Community litracy level**			χ2 = 348.26, *p* < 0.001
Low	3972 (84.78)	713 (15.22)	
Medium	2826 (75.46)	919 (24.54)	
High	1972 (66.53)	992 (33.47)	
**Community socioeconomic level**			χ2 = 258.56, *p* < 0.001
Low	5477 (81.92)	1209 (18.08)	
Medium	1191 (75.05)	396 (24.95)	
High	2102 (67.35)	1019 (32.65)	
**Community level modern contraceptive knowledge**			
Low	3391 (84.00)	646 (16.00)	χ2 = 190.92, *p* < 0.001
Medium	2820 (75.08)	936 (24.92)	
High	2559 (71.06)	1042 (28.94)	

### Factors associated with modern contraceptive use

The results on the individual level factors of modern contraceptive use showed that women’s age (45–49 years-aOR = 0.44, 0.30–0.63), women’s educational level (higher-aOR = 1.88, 1.28–2.76) and husband’s educational level (higher-aOR = 1.43, 1.10–1.85) were associated with  modern contraceptive use among married women in Senegal. Number of living children (5 or more children-aOR = 36.01, 22.73–57.05), ideal number of children (2 children-aOR = 1.95, 1.13–3.35), desire to have more children (wants no more-aOR = 2.46, 2.06–2.94), ethnicity (Diola-aOR = 0.70, 0.50–0.99) and media exposure (yes-aOR = 1.44, 1.16–1.79) also had significant associations with modern contraceptive use among married women in Senegal. Other individual level factors associated with  modern contraceptive use were wealth index (richer-aOR = 1.31, 1.03–1.67) and decision making power of women (decision making two-aOR = 1.20, 1.02–1.41). In relation to the community-level factors, region (Matam-aOR = 0.35, 0.23–0.53), place of residence (rural-aOR = 0.76, 0.63–0.93), community literacy level (high-aOR = 1.31, 1.01–1.71) and community knowledge level of modern contraceptive (high-aOR = 1.37, 1.13–1.67) were found as significant factors (see Model 3 of Table 2).

**Table 2:** Multilevel binary logistic regression results for factors associated with modern contraceptive use among married women in Senegal

Variables	Model 0	Model I	Model II	Model III
**Decision making**				
No decision making (ref)				
Decision making one		1.24 (1.10–1.40)***		1.18 (1.04–1.33)**
Decision making two		1.28 (1.09–1.50)**		1.20 (1.02–1.41)*
**Wife-beating**				
Accept (ref)				
Refuse		1.13 (1.01–1.26)*		1.11 (0.99–1.24)
**Media exposure**				
No (ref)				
Yes		1.54 (1.24–1.91)***		1.44 (1.16–1.79)**
**Women’s educational level**				
No formal education (ref)				
Primary school		1.24 (1.09–1.42)**		1.21 (1.06–1.38)**
Secondary school		1.42 (1.19–1.70)***		1.37 (1.14–1.64)***
Higher		1.98 (1.34–2.92)**		1.88 (1.28–2.76)**
**Religion**				
Muslim (ref)				
Christian		1.08 (0.78–1.50)		1.03 (0.75–1.43)
**Wealth index**				
Poorest (ref)				
Poorer		1.01 (0.86–1.19)		0.97 (0.82–1.14)
Middle		1.41 (1.18–1.69)***		1.20 (0.99–1.46)
Richer		1.72 (1.40–2.11)***		1.31 (1.03–1.67)*
Richest		1.79 (1.40–2.27)***		1.22 (0.91–1.62)
**Women’s age**				
15–19 years (ref)				
20–24 years		1.18 (0.88–1.57)		1.15 (0.87–1.54)
25–29 years		1.10 (0.82–1.47)		1.05 (0.78–1.40)
30–34 years		0.98 (0.72–1.33)		0.94 (0.69–1.28)
35–39 years		0.94 (0.68–1.29)		0.88 (0.64–1.22)
40–44 years		0.70 (0.50–0.98)*		0.66 (0.47–0.92)*
45–49 years		0.47 (0.32–0.68)***		0.44 (0.30–0.63)***
**Number of living children**				
No child (ref)				
1–2 children		12.60 (8.23–19.30)***		12.55 (8.20–19.21)***
3–4 children		24.47 (15.72–38.08)***		24.51 (15.76–38.11)***
5 or more children		35.49 (22.38–56.27)***		36.01 (22.73–57.05)***
**Ethnicity**				
Wolof (ref)				
Poular		0.87 (0.75–1.01)		0.90 (0.77–1.06)
Serer		1.10 (0.91–1.32)		1.08 (0.90–1.31)
Mandingue/soc		1.01 (0.81–1.26)		1.04 (0.82–1.31)
Diola		0.76 (0.55–1.05)		0.70 (0.50–0.99)*
Sonink		0.93 (0.58–1.49)		0.99 (0.62–1.57)
Other Senegalese		0.96 (0.75–1.23)		0.96 (0.74–1.23)
Other		0.68 (0.48–0.98)*		0.66 (0.46–0.95)*
**Husband educational level**				
No formal education (ref)				
Primary school		1.37 (1.17–1.60)***		1.32 (1.13–1.54)***
Secondary school		1.18 (0.99–1.41)		1.14 (0.95–1.36)
Higher		1.45 (1.12–1.88)**		1.43 (1.10–1.85)**
**Ideal number of children**				
0 child (ref)				
1 child		0.79 (0.18–3.41)		0.89 (0.21–3.80)
2 children		1.85 (1.07–3.17)*		1.95 (1.13–3.35)*
3 children		1.07 (0.70–1.66)		1.13 (0.73–1.75)
4 children		1.17 (0.80–1.70)		1.25 (0.86–1.83)
5 children		1.22 (0.84–1.77)		1.31 (0.90–1.90)
6 children and above		1.06 (0.74–1.52)		1.14 (0.79–1.63)
Non-numeric response		0 .80 (0.55–1.18)		0.90 (0.62–1.33)
**Desire for more children**				
Wants within 2 years (ref)				
Wants after 2 years		2.12 (1.85–2.44)***		2.09 (1.81–2.40)***
Wants, unsure timing		0.92 (0.70–1.22)		0.93 (0.70–1.23)
Undecided		1.47 (0.96–2.25)		1.52 (0.99–2.32)
Wants no more		2.50 (2.09–2.99)***		2.46 (2.06–2.94)***
**Distance to health facility**				
Big problem (ref)				
Not a big problem		1.17 (1.02–1.33)*		1.10 (0.97–1.26)
**Region**				
Dakar (ref)				
Ziguinchor			0.60 (0.42–0.86)**	0.69 (0.46–1.04)
Diourbel			0.50 (0.35–0.69)***	0.47 (0.33–0.67)***
Saint Louis			0.73 (0.53–1.00)	0.81 (0.57–1.14)
Tambacounda			0.56 (0.39–0.81)**	0.77 (0.51–1.16)
Kaolack			0.63 (0.46–0.87)**	0.70 (0.50–1.00)
Thies			0.99 (0.74–1.32)	0.96 (0.70–1.32)
Louga			0.67 (0.48–0.93)*	0.75 (0.52–1.07)
Fatick			0.82 (0.59–1.14)	0.75 (0.53–1.08)
Kolda			0.67 (0.48–0.93)*	0.85 (0.59–1.23)
Matam			0.30 (0.21–0.44)***	0.35 (0.23–0.53)***
Kaffrine			0.86 (0.61–1.20)	1.04 (0.72–1.51)
Kedougou			0.37 (0.25–0.57)***	0.51 (0.32–0.80)**
Sedhiou			0.60 (0.43–0.86)**	0.70 (0.48–1.03)
**Place of residence**				
Urban (ref)				
Rural			0.76 (0.63–0.91)**	0.76 (0.63–0.93)**
**Community literacy level**				
Low (ref)				
Medium			1.47 (1.22–1.78)***	1.30 (1.07–1.59)**
High			1.65 (1.29–2.120)***	1.31 (1.01–1.71)*
**Community socioeconomic level**				
Low (ref)				
Medium			1.15 (0.93–1.42)	1.08 (0.86–1.36)
High			1.10 (0.88–1.37)	1.04 (0.81–1.33)
**Community level modern contraceptive knowledge**				
Low (ref)				
Medium			1.37 (1.15–1.62)***	1.34 (1.12–1.61)***
High			1.46 (1.22–1.76)***	1.37 (1.13–1.67)***
**Random effect result**				
PSU variance (95% CI)	0.52 (0.42–0.65)	0.30 (0.23–0.40)	0.16 (0.11–0.22)	0.16 (0.11–0.24)
ICC	0.13	0.08	0.04	0.04
LR Test	X^2^ = 443.55, p < 0.001	X^2^ = 152.76, p < 0.001	X^2^ = 76.02, p < 0.001	X^2^ = 59.26, p < 0.001
Wald chi-square and p-value		X^2^ = 883.97, p < 0.001	X^2^ = 343.82, p < 0.001	X^2^ = 1028.03, p < 0.001
**Model fitness**				
Log-likelihood	− 5926.8	− 5113.3	− 5793.98	−5048.23
AIC	11,857.64	10,316.66	11,631.96	10,226.48
BIC	11,872.32	10,645.76	11,793.46	10,701.84
PSU	400	400	400	400

Notes: *AOR* Adjusted odds ratios, *COR* Crude odds ratio *** *p* < 0.001, ** *p* < 0.01, * *p* < 0.05. Ref = reference

In terms of model fitness, the complete model (Model 3), which included all the individual and community level factors had the highest AIC of 10,226.48 and the lowest log-likelihood ratio of − 5048.23 and was thus considered as the best fit model for predicting the occurrence of modern contraceptive use among married women.

## Discussion

Our study examined modern contraceptive use and its associated factors among married women in Senegal. Age, women’s and husband’s level of education, women’s decision making capacity, exposure to media, number of living children, desire to have more children, ideal number of children, place of residence, ethnicity, community literacy level, community level modern contraceptive knowledge and region were associated with modern contraceptive use.

In line with previous studies [47, 48], our findings showed that the use of modern contraceptive decreases with age, controlling for other factors. We observed that the use of modern contraceptives was high among younger women compared to older women. This higher uptake among younger women has been attributed to effective communication on family planning matters with their husbands/partners [49].

We found that women’s educational level influences uptake of modern contraceptive use, with better utilization among women with higher educational level, which is supported by a previous study [49]. As the level of education increases, wealth and prestige tend to increase and the intention to limit children by using modern contraceptives will increase [50]. Apparently, education leads to a greater ability to acquire wealth and prestige; this competes with decision on childbearing since in a modern economy children are for many years resource consumers rather than resource producers [50]. The findings indicate the need to consider education during awareness creation such as using audio-visual aid instead of leaflets distribution.

The study further revealed that utilization of modern contraceptives is connected to partner’s educational level, and media exposure. The odds of use of modern contraceptives among married women whose partners had completed at least primary education were higher compared to those whose partners had no formal education. This is consistent with the findings of previous studies [13, 20] that have shown that partners/husbands with higher levels of education may support women’s contraceptive usage through high levels od communication [20, 21]. Relatedly, women who had exposure to newspaper, radio or television for at least once a week had higher likelihood of using modern contraceptive methods than women with no media exposure. Mass media has been considered as a powerful tool that can increase the demand for health services as it provides individuals within a community the means to identify, respond to, and address their own needs [22]. The media may also be beneficial in raising awareness on health issues within communities, as well as addressing social and cultural issues. This crucial role of the media can help reduce the barriers to access and use of health services including contraceptive services [23].

Consistent with findings from prior studies [24–26], we further found that the odds of modern contraceptive use was higher among richer women as compared to poorest. This could be due to wealth influence, related to their socioeconomic status including access to modern health care and education [25, 26]. We further found a significant association between the number of living children and the use of modern contraceptive, with married women with living children more likely to use modern contraceptives compared to those with no living children. Similar findings have been obtained in previous studies [6, 27, 28].

Living in urban areas was found to lead to a higher likelihood of using modern contraceptive methods than in rural areas, consistent with previous studies [17, 29]. This could be due to the fact that urban residents are less likely to travel long distances for medical services than their rural counterparts [59]. Furthermore, not all health facilities are equipped with contraceptive methods, particularly in Southern regions [59, 60]. According to previous studies, a national intervention aimed at strengthening the contraceptive supply chain had not been implemented in the southern part of the country [17, 60]. Available evidence in Senegal [17] suggested that knowledge about contraceptive methods is highly associated with uptake and it has been shown that those living in rural areas may have little knowledge on modern contraceptive methods [17]. In Senegal, low contraceptive demand among rural women may be due to geographical and financial barriers associated with access to contraceptive services [61], as well as reluctance to use family services [62].

Similarly, we observed differences in the utilization of modern contraceptive across regions, consistent with previous findings [17]. Modern contraceptives use was identified to be lower in almost all the regions as compared to Dakar region. This finding can be attributed to the existence of family planning services, and the increasing number of health facilities in urban areas [13]. Prior studies in Senegal noted an unequal repartition of health structures, and health personnel across regions with higher absorptions and accumulations of potentialities in few regions such as Dakar regions as major concern for creating disparities across regions [13, 17, 35, 60].

A significant association was found between ethnicity and contraceptive utilization as seen in a prior study [30]. This phenomenon may be attributed to the unequal access of public services to people lower in the ethnic hierarchy, illiteracy, and poor socio-economic conditions among some ethnic groups. Other reasons for the disparities in modern contraceptive use across ethnic groups could be the health insurance use, attitudes of physicians towards minority people, language barriers, transportation cost, low level of education, illiteracy, poverty and low socio-economic status, familiarity with the health care delivery system, the degree and kind of family support [31–33].

In line with a previous study [18], we found better uptake of modern contraceptives among married women with decision making power as compared to married women who had no decision making power. Beside increasing uptake of contraceptive methods, empowering women has an imperative role in the decrease of maternal and new born mortality by preventing unplanned pregnancy and unsafe abortion [18]. We also found that community-level literacy and community knowledge of modern contraceptives affect the utilization of modern contraceptive. Education can empower societies including women through improving their independence and involvement in decision-making, absolutely transforming health-seeking behavior and building social capital through development of social networks [59].

### Strengths and limitation of the study

The key strength of this study lies in the use of nationally representative DHS data and a large sample size. Nonetheless, some study limitations were also observed. The study design (cross-sectional) does not give us the opportunity to establish causality between variables. Second, since we used secondary data, other determinants such as quality of care including counseling about side effects and accessibility of supply, and cultural factors were not included in the models. To deal with the limitations associated with the cross-sectional design and the absence of some essential variables in this study, a longitudinal study that uses primary data and gathers information on all relevant factors associated with the use of modern contraceptives is recommended.

## Conclusion

Utilization of modern contraceptives may be influenced by both individual and community-level factors. While, women’s age, women and husband’s educational level, media exposure, number of living children, desire to have more children, ideal number of children, economic status, women decision making power, ethnicity were the individual-level factors, place of residence, community literacy level, community level modern contraceptive knowledge and subnational region were found to be the important community-level determinants for the utilization of modern contraceptive methods in this study. Therefore, interventions should focus on enhancing literacy levels, for both the women and their husbands. Additionally, to satisfay the demand of disadvantaged women (uneducated, rural residents, poor, and those living in some regions), using opportunities like mini media promotion campaign in a place where those women are available including market day could be considered. Moreover, integrating contraceptive service (offer) with child health service such as child Out Patient Department (OPD), immunization service, could be in fixed or mobile vaccination site as well as with maternal service such as couple counseling about contraceptive during ANC care and offering the service during PNC and post abortion time. Furthermore, strengthening awareness and attitude towards family planning should be given priority, in addition to designing intervention to increase accessibility and affordability of contraceptive methods especially in rural areas and regions with low resources. Finally, empowering women economically and through education can be helpful.

## Data Availability

The datasets generated and/or analyzed during the current study are available in http://dhsprogram.com/data/available-datasets.cfm.

## Abbreviations

ANC: Antenatal care
AOR: Adjusted Odd Ratio
CI: Confidence Interval
C-DHS: Continuous Demographic and Health Survey
COR: Crude Odd Ratio
DHS: Demographic and Health Survey
EA: Enumeration Area
ICF: Inner City Fund
IR: Individual Recode
IRB: Institutional Review Board
PNC: Posnatal care
PPS: Probability Proportional to Size
PSU: Primary Sampling Unit
SDG: Sustainable Development Goal
WHO: World Health Organization
